# eIF2α Phosphorylation by GCN2 Is Induced in the Presence of Chitin and Plays an Important Role in Plant Defense against *B. cinerea* Infection

**DOI:** 10.3390/ijms21197335

**Published:** 2020-10-04

**Authors:** Marta Berrocal-Lobo, René Toribio, M. Mar Castellano

**Affiliations:** 1 Centro de Biotecnología y Genómica de Plantas, Universidad Politécnica de Madrid (UPM)—Instituto Nacional de Investigación y Tecnología Agraria y Alimentaria (INIA), Campus de Montegancedo UPM, 28223 Pozuelo de Alarcón, Madrid, Spain; toribio.francisco@upm.es; 2 Departamento de Sistemas y Recursos Naturales, E.T.S.I. Montes, Forestal y del Medio Natural, Ciudad Universitaria s/n, 28040 Madrid, Spain

**Keywords:** GCN2, eIF2α phosphorylation, chitin, *B. cinerea*, necrotrophic fungi, translation regulation, defense-related genes

## Abstract

Translation plays an important role in plant adaptation to different abiotic and biotic stresses; however, the mechanisms involved in translational regulation during each specific response and their effect in translation are poorly understood in plants. In this work, we show that GCN2 promotes eIF2α phosphorylation upon contact with *Botrytis cinerea* spores, and that this phosphorylation is required for the proper establishment of plant defense against the fungus. In fact, independent *gcn2* mutants display an enhanced susceptibility to *B. cinerea* infection, which is highlighted by an increased cell death and reduced expression of ethylene- and jasmonic-related genes in the *gcn2* mutants. eIF2α phosphorylation is not only triggered in the presence of the fungus, but interestingly, is also achieved in the sole presence of the microbe-associated molecular pattern (MAMP) chitin. Moreover, analysis of de novo protein synthesis by ^35S^Met–^35S^Cys incorporation indicates that chitin treatment promotes a global inhibition of translation. Taken together, these results suggest that eIF2α phosphorylation by GCN2 is promoted in the presence of chitin and plays an important role in plant defense against *B. cinerea* infection.

## 1. Introduction

Translation is highly regulated in plants during different developmental programs and in response to multiple stimuli [1,2,3]. However, despite the well-known relevance of regulation of translation in these eukaryotes, the mechanisms involved in translational control in the plant kingdom remain mainly unknown. This lack of knowledge is revealed by the observation that some important translational regulators in mammals and fungi are missing in plants and some others that seem to be conserved show a different level of specialization [4,5]. 

In other eukaryotes, one of the best-known mechanisms for translational regulation is mediated by eukaryotic initiation factor 2 subunit α (eIF2α) phosphorylation. This phosphorylation inhibits the conversion of eIF2-GDP to eIF2-GTP, blocking further cycles of translation initiation [6]. Among the different eIF2α kinases described in eukaryotes, GCN2 (for general control non-derepressible 2) is the only eIF2α kinase described in plants [2,4,5,7,8,9]. In other eukaryotes GCN2 has a prominent role in amino acid starvation response [10], and in accordance to this role in plants, AtGCN2 complements the *gcn2* yeast mutant strain in the presence of inhibitors of branched-chain and histidine amino acid biosynthesis [11]. In addition, AtGCN2 also phosphorylates eIF2α during the treatment with herbicides that interfere with amino acid biosynthesis such as chlorsulfuron and glyphosate [8,12]. Furthermore, in vitro analysis using purified proteins revealed the ability of AtGCN2 to bind uncharged tRNA and to phosphorylate AteIF2α [13]. All together, these results suggest that AtGCN2 directly phosphorylates AteIF2α and highlight the conserved role of GCN2 as a sensor of amino acid availability in plants.

Plant GCN2 has also been involved in other specific developmental programs, abiotic stress responses, and defense [7,14,15]. In this latter sense, it has been described that GCN2-mediated eIF2α phosphorylation is induced by wounding and by hormones involved in plant defense such as salicylic acid (SA), jasmonic acid (JA), methyl jasmonate (MeJA), and the ethylene (ET) precursor 1-aminocyclopropane-1-carboxylic acid (ACC) [7,14]. In addition, GCN2-mediated eIF2α-P is induced by the presence of the whitefly *Bemisia tabaci* in tobacco plants [14] and in response to bacterial infection by *P. syringae pv. tomato* (Pst) strains DC3000 and DC3118 in Arabidopsis [16]. Despite these pieces of evidence suggesting a role of GCN2 and eIF2α phosphorylation in plant defense, it is still unclear whether eIF2α phosphorylation can play a main role in other pathogen responses and which are the molecular players involved in the activation of the GCN2 pathway. This produces a gap in the knowledge on the possible effect of eIF2α in the regulation of translation during plant immunity.

## 2. Results

### 2.1. gcn2 Mutants Show Impaired Defense Response against Botrytis cinerea

**Figure 1:**
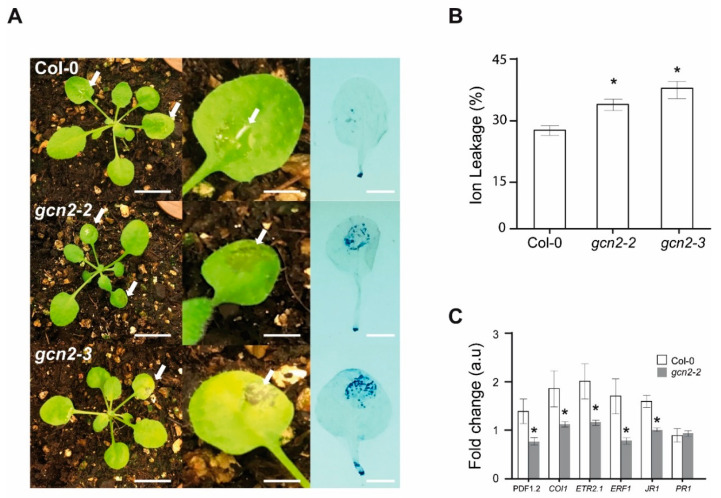
AtGCN2 participates in the defense response to *Botrytis cinerea*. (**A**) Representative disease symptoms in 3-week-old seedlings from Col-0 (WT) and *gcn2-2* (SALKseq_032196) and *gcn2-3* (SALKseq_129334) mutants at 3 dpi (right panel). For each experiment, two leaves from each plant were inoculated with 5 µL of a *Botrytis* suspension (5 × 10^6^ spores/mL). Close-up views of a representative *B. cinerea* infected leaf from each genotype (middle panel). In these two panels, lesions are highlighted with white arrows. Representative cell death symptoms on leaves detected by Trypan blue staining (right panel). Scale bars correspond to 1 cm in the left panel and 0.25 cm in the middle and right panels. These experiments were repeated independently at least three times, obtaining similar results. (**B**) Analysis of ion leakage from infected leaves of the same lines at 3 dpi. The percentage of ion leakage upon *Botrytis* inoculation is related to the total conductivity of the leaves after membrane and wall breakdown. *n* = 6 independent experiments including 10 leaves per genotype. Statistical differences (* *p* ≤ 0.05) are highlighted by asterisks. (**C**) qRT-PCR analysis of *AtPDF1.2*, *AtCOI1*, *AtETR2.1*, *AtERF1*, *AtJR1,* and *AtPR1* transcription levels at 1 dpi. Fold change expression is related to the expression of each gene in the corresponding genotype under control conditions (i.e., in the absence of fungus), which was arbitrarily assigned value 1 after normalization to *β-ACTIN* (internal normalization control). In this case, three biological replicates were analyzed. For (B and C), values are shown as mean ± SEM. In both cases, statistically significant differences (* *p* < 0.01) were calculated using one-way ANOVA test.

In order to analyze if GCN2 plays a role during the plant defense response to *B. cinerea,* two independent Col-0 Arabidopsis *gcn2* mutants, *gcn2-2* (SALKseq_032196) and *gcn2-3* (SALKseq_129334) were inoculated with *B. cinerea*. As shown in Figure 1A, compared to the wild-type plants (Col-0), both *gcn2* mutants showed severer necrotic lesions and chlorosis symptoms upon infection with the fungus. In addition, they showed a higher level of cell death on the infected leaves (analyzed by Trypan blue staining). These symptoms were correlated with an increased ion leakage in the *gcn2* mutant lines (Figure 1B).

It is well-known that ET and JA pathways play a main role in plant defense against *B. cinerea* infection [17,18,19]; therefore, to determine if this higher susceptibility is also reflected at the molecular level, we carried out qRT-PCR analysis of ET and JA marker genes. Since both mutants showed a similar phenotype, we selected for these experiments one of the *gcn2* mutant lines. As shown in Figure 1C, compared to the wild-type plants, with the only exception of *PR1*, the induction in the expression of the other five ET and JA marker genes was reduced in the *gcn2-2* mutant upon *B. cinerea* contact. These results suggest that the defense transcriptional response to *B. cinerea* is not fully accomplished in *gcn2* mutants.

Altogether, the results indicate that GCN2 is involved in plant immunity against the necrotrophic fungus *B. cinerea*. These results are in accordance with the reduced induction of ET- and JA-related mRNAs in the *gcn2* mutant analyzed by (Faus et al., 2015) and with the high production of MeJA and the lower accumulation of SA in the NtGCN2 overexpressing plants [14].

### 2.2. eIF2α Is Phosphorylated upon Plant Contact with B. cinerea Spores and This Phosphorylation Is Dependent on GCN2

**Figure 2:**
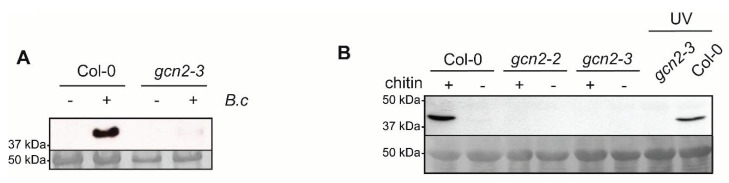
GCN2-dependent eIF2α phosphorylation is induced upon contact with *B. cinerea* spores and by chitin. (**A**) Analysis of eIF2α-P of wild-type (Col-0) and *gcn2-3* (SALKseq_129334) seedlings mock inoculated (-) or incubated with a suspension of 5 × 10^6^ spores/mL of *Botrytis cinerea (B.c) (+)* for 60 min. (**B**) Analysis of eIF2α phosphorylation of wild-type (Col-0), *gcn2-2* (SALKseq_032196) and *gcn2-3* (SALKseq_129334) seedlings incubated in the presence of 400 mg/L chitin (+) or in the absence of chitin (−) for 1 h. As control, Col-0 seedlings were subjected to an UV treatment known to promote eIF2α phosphorylation. In all cases, 7-day-old seedlings were used for the experiments and eIF2α phosphorylation was analyzed by Western blot (upper panels). As loading control of each experiment, representative bands from the Coomassie staining of the original membrane are provided (lower panels). All the experiments were independently repeated at least three times obtaining similar results.

Since the results described above suggest that GCN2 is involved in plant defense against *B. cinerea*, and GCN2 has been described as an eIF2α kinase in plants [11], we decided to study if GCN2 promotes eIF2α phosphorylation during incubation with the fungus. To do so, seedlings from Col-0 wild-type genotype and the *gcn2-3* mutant were incubated with *B. cinerea* spores. Subsequently, the incubated plants were collected, and eIF2α phosphorylation was detected by Western blot using an anti-phospho-eIF2α antibody. As shown in Figure 2A, compared to the mock treated plants, eIF2α phosphorylation was prompted in plants upon contact with *B. cinerea*. This phosphorylation was dependent on GCN2, since no phosphorylation was observed in the *gcn2* mutant.

### 2.3. Chitin Induces GCN2-Dependent eIF2α Phosphorylation

One of the first processes that takes place on the plant surface during the establishment of the defense is the recognition of the MAMP chitin of the fungus [20]; therefore, in order to analyze if eIF2α phosphorylation is triggered upon fungus recognition, we decided to study eIF2α phosphorylation in response to chitin. For this, Col-0 Arabidopsis seedlings were incubated in the presence or absence of chitin and eIF2α phosphorylation was monitored by Western blot (Figure 2B, left part). In parallel, we also analyzed chitin-triggered phosphorylation in the *gcn2-2* and *gcn2-3* mutants (Figure 2B, middle part). As positive controls of eIF2α phosphorylation, we included a treatment with UV light, a well-known agent that promotes eIF2α phosphorylation in plants [7] (Figure 2B, right part). As shown in Figure 2B, as it was also the case of the UV treatment, chitin-promoted eIF2α-phosphorylation and this phosphorylation was fully abolished in the *gcn2-2* and *gcn2-3* mutants. All these data highly suggest that chitin prompts eIF2α phosphorylation in plants and that this phosphorylation is dependent on GCN2.

To provide further evidence on the possible conservation of this phosphorylation in a different ecotype, we decided to analyze if chitin also promotes eIF2α phosphorylation in the Ler genotype, and for this, we took advantage of the previously described *gcn2* mutant GT8359 [8]. As observed in Supplementary Figure S1, we also observed a GCN2-dependent phosphorylation by chitin in this different background, which further reinforces that this mechanism is conserved in different Arabidopsis ecotypes.

### 2.4. Chitin Promotes a Global Reduction of Translation

It is well-known that eIF2α phosphorylation leads to a global inhibition in protein translation in mammals and yeast [10]. However, despite multiple pieces of evidence indicating that eIF2α phosphorylation is induced in plants in response to different stresses, including upon SA, JA, and ACC treatments [7], whether this phosphorylation is associated to global changes in translation has remain elusive, except in the case of treatments with chlorsulfuron and 8-azaadeninine (AZA), which course with an accumulation of uncharged tRNAs.

**Figure 3:**
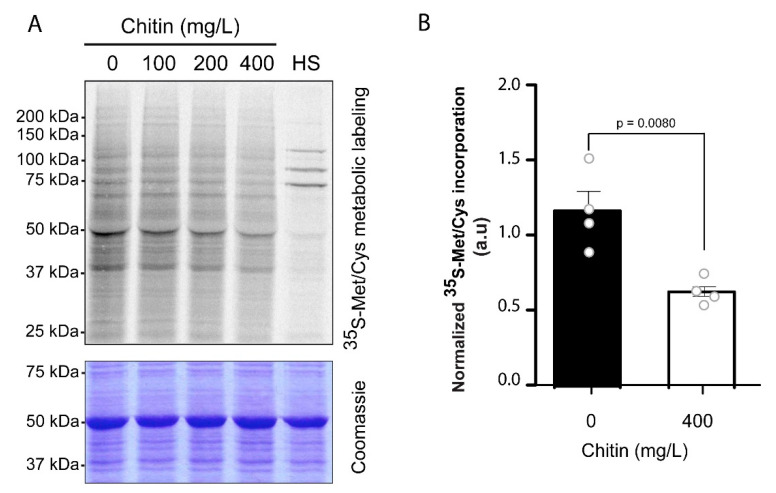
Chitin triggers a global reduction in protein synthesis. (**A**) Metabolic labeling of de novo synthesized proteins. Arabidopsis 7-day-old seedlings were incubated with ^35^S-Methionine ^35^S-Cysteine in the absence of chitin (-) or in the presence of different chitin concentrations (upper panel). As positive control of global inhibition of protein synthesis, seedlings were treated at 38 °C for 30 min (HS). The Coomassie staining of the gel is provided as loading control (lower panel). This experiment was repeated at least three times obtaining similar results. (**B**) Normalized quantification of the ^35^S-Methionine ^35^S-Cysteine incorporation into proteins (related to the total amount of loaded protein per lane) in the absence (-) or in the presence of chitin (400 mg/L). The bars show the mean ± SEM of *n* = 4 independent experiments. Statistically significant differences (*p* < 0.008) were calculated by *t*-student test.

Thus, in order to investigate whether chitin perception leads to a global effect on plant translation, Arabidopsis seedlings were treated with different concentrations of chitin, and subsequently, the seedlings were labeled with ^35^S-Methionine ^35^S-Cysteine. After labeling, equal amounts of total protein were loaded in a gel and run. The gel was subjected to Coomassie staining (Figure 3A, lower panel, provided as loading control) and ^35^S-Methionine ^35^S-Cysteine incorporation into the de novo synthesized proteins was analyzed by autoradiography (Figure 3A, upper panel). As positive control, we also monitored protein synthesis in seedlings subjected to heat stress, a treatment that promotes a drastic inhibition of protein translation [1]. As shown in Figure 3A, upper panel, chitin induced a reduction in the global protein synthesis that was enhanced at higher concentrations of chitin. In the case of the treatments carried out at 400 mg/L chitin, the reduction in de novo protein synthesis was quantified (Figure 3B). This analysis indicates that chitin promotes a significant reduction in the de novo protein synthesis. 

In order to corroborate the chitin-induced reduction in protein synthesis by a different technique, we also carried out polysome profile analyses. To do so, extracts from Arabidopsis seedlings incubated in the absence or presence of 400 mg/L chitin were subjected to an ultracentrifugation in a 10–50% sucrose gradient. This technique allows the separation of mRNAs by density according to the number of ribosomes they are associated with. In such a way, those RNAs associated to multiple ribosomes, and so active in translation, migrate to the heavier fractions of the gradient that are called polysomal fractions. As shown in Supplementary Figure S2, compared to the control conditions, a modest decrease in polysomal fractions was observed in the chitin-treated plants, providing an alternative indication of the global inhibition of translation upon chitin treatments. All these results suggest that chitin triggers a GCN2-dependent eIF2α-phosphorylation in plants and a global reduction in protein synthesis.

## 3. Discussion

Translation has recently emerged as a main mechanism to regulate gene expression during plant development and, especially, under environmental challenges [3,4,5,9]. Translation consumes a substantial amount of cellular energy, and so, upon environmental challenges, translation should be adjusted to reduce the consumption of energy while allowing the translation of key proteins involved in plant adaptation to stress. This is achieved by the establishment of a global translation inhibition and the selective synthesis of key stress regulators. Despite the output of this regulation being well-established, the knowledge about how this translational regulation is achieved, and the targets of this regulation is extremely scarce in the plant kingdom compared to other eukaryotes [2,4,5,9]. 

Constitutive plant immunity involves the activation of basal defense after recognition of pathogen/microbe-associated molecular patterns (PAMPs/MAMPs) by pattern-recognition receptors (PRRs) [21]. MAMPs embrace a wide diversity of conserved molecules across a wide range of microbes, being pathogenic or not. Their recognition contributes to the plant basal resistance through the activation of pathogen-triggered immunity (PTI), during compatible and non-compatible interactions. An example of PAMPs is chitin (a β-linked sugar polymer that is present in the structures of fungal spores, yeast cell walls, insect exoskeletons, and shells of arthropods and invertebrates) [22].

Until now, different studies demonstrated that GCN2 is involved in eIF2α phosphorylation in response to other pathogens and to different phytohormones associated to plant immunity [7,14,15]. In this study, we report that GCN2 is also involved in plant defense against *B. cinerea* infection. This conclusion is supported by the higher susceptibility to this fungus of the *gcn2* mutants, which is accompanied by a reduced expression of genes involved in different steps of the ET- and JA-signaling pathways. Furthermore, this is the first time that the role of a MAMP (and specifically chitin) in eIF2α phosphorylation and in the regulation of global translation in plants has been addressed. Although the effect of chitin in protein translation could be observed at lower concentration of chitin (100 and 200 mg/L, Figure 3), the strongest effect in inhibition of protein synthesis is achieved at 400 mg/L chitin, which prompted us to select this concentration for our experiments. This concentration, although higher than the used in normal basis (100 mg/L), is in the range of the chitin concentrations proven to produce effects related to defense in plants (such as callose deposition) [23]. Furthermore, the phosphorylation observed in Ler background in response to 100 and 200 mg/L chitin strongly suggests that this phosphorylation could be achieved in a lower range of chitin concentrations (Figure S1).

**Figure 4:**
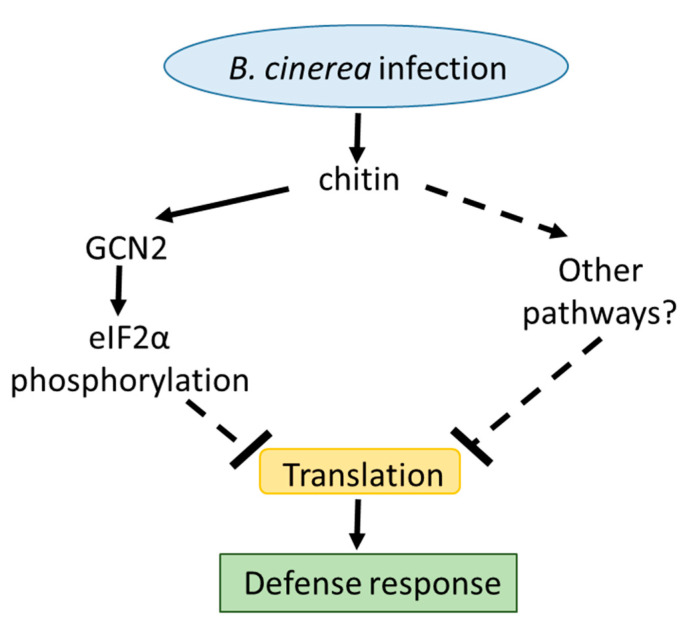
Proposed model for translation inhibition in response to chitin. Our results strongly suggest that chitin perception induces translation inhibition in plants. Chitin promotes, via activation of GCN2, the phosphorylation of eIF2α. This mechanism may contribute, most probably in concert with others, to translation inhibition in response to this signaling molecule. This inhibition of translation, most probably along with the selective translation of proteins involved in coping with the stress, may contribute to the proper activation of plant defense. The main role of regulation of translation in plant immunity has also been highlighted by other authors such as [24,25]. Solid and dashed lines represent proven and unknown connections among the processes, respectively.

As observed, the reduction in global translation induced by chitin is moderate, especially when compared with the extreme global translation induced by heat stress. In this sense, it has to be considered that translation constitutes a fundamental step in gene expression, and therefore, changes in global translation should be finely controlled to allow a precise adaptation response to the environment. Accordingly, it seems logical that the effect (in quantitative terms) on translatome remodeling depends on the severity of the stress and on the damage induced by it. Our translation experiments were carried out in response to chitin treatments. Under such conditions, it is not expected that plants are subjected to a severe stress (as compared to heat), but to a priming process that may be achieved with more moderate changes in global translatome. Whether eIF2α phosphorylation is the sole trigger of this translational inhibition seems improbable. It should be considered that there are multiple mechanisms of translation inhibition for assuring proper gene expression and energy saving under stress conditions, and eIF2α phosphorylation is just one of them. In this sense, it is possible that other mechanisms of translation regulation could be involved and show different degree of influence in this translational control (Figure 4).

## 4. Materials and Methods

### 4.1. Plant Material and Growth Conditions

For in vitro growth, Arabidopsis seeds were surface sterilized using 0.05% (*v*/*v*) Tween-20 and 80% (*v*/*v*) ethanol for 5 min and stratified at 4 °C in darkness for 48 h. The sterilized seeds were germinated on Murashige and Skoog (MS) medium (Duchefa, Haarlem, The Netherlands) supplemented with 1% (*w*/*v*) sucrose and 9 g/L of plant agar. The plates were maintained in vertical position under long-day photoperiodic conditions (16/8 h light/dark schedule) at 22 °C constant temperature. For growth in soil, Arabidopsis plants were grown in a mixture of soil-vermiculite (3:1) under long-day photoperiodic conditions at 22 °C day/19 °C night temperature, 65% relative humidity, and 120 μEm^−2^s^−1^ of light intensity.

The *gcn2* mutant lines GT8359 (*Landsberg erecta* background), SALKseq_032196 and SALKseq_129334 (these two latter lines in Col-0 background) were obtained from the NASC (Nottingham Arabidopsis Stock Centre, available online http://arabidopsis.info/BasicForm). When obtained, the lines SALKseq_032196 and SALKseq_129334 contained, in addition to a T-DNA insertion in the *GCN2* gene, other T-DNA insertions in other parts of the genome, respectively. These lines were crossed to Col-0 and segregated until no amplification of the T-DNA sequences flanking the additional insertions were obtained in the *gcn2* homozygous mutants. During the course of this work, the lines SALKseq_032196 and SALKseq_129334 were described and named as *gcn2-2* and *gcn2-3* [26,27], respectively. This nomenclature has been maintained in the text and figures of the article.

### 4.2. Phytopathogens, Storage, and Growth Conditions

The fungal pathogen *Botrytis cinerea* [28] was grown on potato dextrose agar medium (PDA) at 28 °C for 8 days, as previously described in [17]. Spores were collected in sterile water, filtered, quantified with a Neubauer chamber, and stored in 20% glycerol at −80 °C until use. 

### 4.3. Pathogen Inoculation and Phenotypic Assessment

Three-week-old Abidopsis plants were used for pathogen inoculation experiments. For phenotypic assessment plant leaf surfaces were inoculated with 5 μL of *B. cinerea* fungal inoculum (5 × 10^6^ spores/mL) in potato dextrose broth medium (PDB). Inoculated plants were placed in a growth chamber allowing fungal growth until harvesting. Cell death was determined by Trypan blue staining after 3 days post inoculation (dpi), as described in [29]. In the case of ion leakage analysis, inoculated leaves were harvested 3 dpi and incubated in water for 4 h, when the initial conductivity of the solution was measured. This value was normalized to the total ion content of the analyzed leaves (obtained by the analysis of the total conductivity of the solution after membrane and wall breakdown by incubation at −80 °C). Six independent replicates were assayed, each of them included at least 10 leaves per genotype. Significant differences were calculated using ANOVA test and the Statgraphics^®^ program (version Centurion XV), Statgraphics Technologies, Inc., The Plains, Virginia, USA.

### 4.4. Gene Expression Analysis by Quantitative Real-Time PCR (qRT-PCR)

Four leaves from 4 different inoculated plants per genotype were harvested at 1 dpi. RNA isolation was carried out using the TRIzol reagent (Thermo Fisher). qRT-PCR analysis was performed as described in [30], using 1 µg RNA for cDNA synthesis and *β-ACTIN* (At3g18780) for internal normalization. Fold change expression was related to the expression of the gene in the corresponding genotype under control conditions (i.e., in the absence of fungus), which was arbitrarily assigned value 1 after normalization to *β-actin*. Three biological replicates, each of them including three technical replicates, were analyzed. Primer sequences are listed in Supplementary Table S1. Statistically significant differences were calculated using paired Student´s t-test using the analysis tool Statgraphics^®^ (version Centurion XV program), Statgraphics Technologies, Inc., The Plains, Virginia, USA. 

### 4.5. eIF2α Phosphorylation Analysis

For the analysis of eIF2α phosphorylation in response to *Botrytis cinerea,* 10 seedlings were transferred to tubes containing *B. cinerea* spores in liquid MS medium. Controls were incubated in a similar solution in absence of the fungus. For the analysis of eIF2α phosphorylation in response to chitin, five to ten 7-day-old Arabidopsis seedlings (grown in vertical position) were transferred to a 24-well-plate and incubated for 1 h at 20°C in 1.2 ml of either MiliQ water (control conditions) or MiliQ water supplemented with 400 mg/L chitin from shrimp shells (SIGMA # C9752), previously autoclaved for 20 min to enhance solubilization. As controls of eIF2α phosphorylation, the seedlings were exposed to UV-C radiation [7,8]). After the treatments, seedlings were quickly frozen in liquid nitrogen, ground to powder, and solubilized in Laemmli sample buffer. An amount of 20 μg of protein extracts were loaded into 12% SDS-PAGE gels, blotted to nitrocellulose membranes, and analyzed with a specific phosphoantibody that recognizes the Ser51 eIF2α-phosphorylated form (Ref:9721, Cell Signaling, 1/1000 dilution). 

### 4.6. Metabolic Labeling of Newly Synthesized Proteins

Labeling of newly synthesized proteins was carried out as described in [1] with the following modifications: 7-day-old seedlings subjected to a 38°C heat stress treatment for 45 min (HS control) or treated with different chitin concentrations were supplemented with 50 µCi/mL ^35S^Methionine/^35S^Cysteine (EasyTagTM EXPRESS35S Protein Labeling Mix, Perkin Elmer, Waltham, MA, USA) for 15 min. Seedlings were washed three times with water, quickly frozen in liquid nitrogen, ground to powder, and solubilized in 1x Laemmli sample buffer. Equivalent amounts of plant extracts were subjected to 10% SDS-PAGE electrophoresis, and ^35^S-labeled proteins were detected by autoradiography using Fuji BAS-SR type image plates and a Typhoon scanner. Coomassie staining of the gels was carried out and used as protein loading controls. Intensities of radiolabeled and Coomassie lanes from the gels were estimated using ImageJ software [31]. In each case, the intensity of the label of ^35S^ Met ^35S^Cys was normalized to the intensity of each loading control. Four independent biological replicates were used for quantification. Statistically significant differences were calculated by Student’s *t*-test.

### 4.7. Polysome Profiling

Seven-day-old seedlings treated in the absence or presence of 400 mg/L chitin were harvested and immediately frozen in liquid nitrogen. An amount of 700 mg of frozen tissue were pulverized in liquid nitrogen with a mortar and pestle. Polysome analysis was carried out as described in [32].

## Supplementary Materials

Supplementary materials can be found at https://www.mdpi.com/1422-0067/21/19/7335/s1. Supplementary Table S1. Primers used in this study for PCR and real time qRT-PCR analysis. Supplementary Figure S1. Analysis of eIF2α phosphorylation of wild-type (Ler) and *gcn2* (GT8359) seedlings incubated in the absence of chitin (-) or in the presence of different concentrations of chitin for 1 h. Seven-day-old seedlings were used for the experiments, and eIF2α phosphorylation was analyzed by Western blot (upper panel). As loading control, representative bands from the Coomassie staining of the original membrane are provided. These experiments were repeated at least three times with similar results. Supplementary Figure S2. Polysome profiles of 7-day-old seedlings from Col-0 incubated for 1 h in the absence of chitin or in the presence of 400 mg/L chitin. The experiment was repeated three times.

## Abbreviations

GCN2	general control non-derepressible 2
eIF2α	eukaryotic initiation factor 2 subunit α
SA	salicylic acid
JA	jasmonic acid
MeJA	methyl jasmonate
ET	ethylene
ACC	1-aminocyclopropane-1-carboxylic acid
