# Supplementary material for: eIF2α Phosphorylation by GCN2 Is Induced in the Presence of Chitin and Plays an Important Role in Plant Defense against B. cinerea Infection
Source: Int J Mol Sci. 2020 Oct 4;21(19):7335. doi: 10.3390/ijms21197335 (PMC7582497; doi:10.3390/ijms21197335)
Supplement: Supplementary file 1 [file ijms-21-07335-s001.zip › sup table1.pdf]

Supplementary Table 1

Oligo Name	Sequence (5'-3')	Gene
PR1F	GATAGCCCACAAGATTATCGG	*PR1* (At2g14610)
PR1R	CTCGTTCACATAATTCCCACG	*PR1* (At2g14610)
PDF1.2F	GGTGGAAGCACAGAAGTTG	*PDF1.2* (At5g44420*)*
PDF1.2R	GCTGGGAAGACATAGTTGC	*PDF1.2* (At5g44420*)*
ETR2.1F	GGTGTTTGTAAGAAAGTGGTG	*ETR2.1* (AT3G23150*)*
ETR2.1R	GTCAGGAGCTGGCGACTC	*ETR2.1* (AT3G23150*)*
ERF1F	CCGATCAAATCCGTAAGC	*ERF1* (AT3G23240)
ERF1R	CGAGCCAAACCCTAATACC	*ERF1* (AT3G23240)
C0I1F	GTGTCCTAATTTGGAAGTTCTC	*COI1* (AT2G39940)
COI1R	CTAAGCCTTCTTCGTCC	*COI1* (AT2G39940)
ACTINF	GTTGGTGATGAAGCACAATCCAAG	*ACTIN2 (* At3gl8780*)*
ACTINR	CTGGAACAAGACTTCTGGGCATCT	*ACTIN2 (* At3gl8780*)*
