# Supplementary figures and images for: eIF2α Phosphorylation by GCN2 Is Induced in the Presence of Chitin and Plays an Important Role in Plant Defense against B. cinerea Infection
Source: Int J Mol Sci. 2020 Oct 4;21(19):7335. doi: 10.3390/ijms21197335 (PMC7582497; doi:10.3390/ijms21197335)

Supplementary figure 1

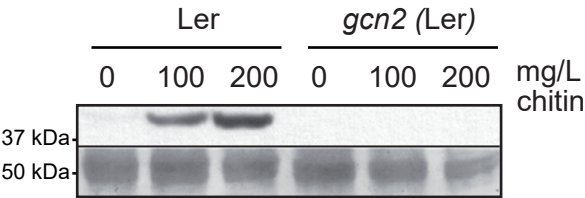

Supplement: Supplementary file 1 [file ijms-21-07335-s001.zip › Supp_Figure1_ijms.pdf]

Supplementary figure 2

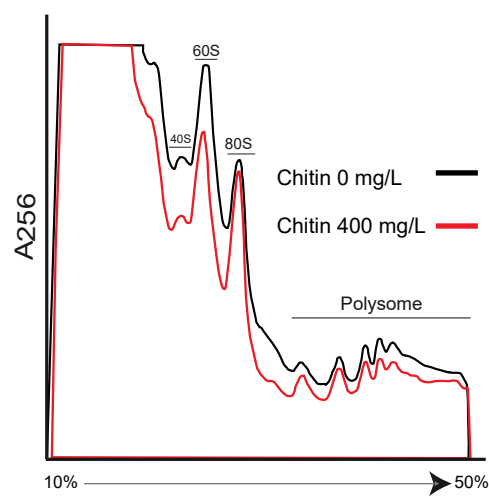

Supplement: Supplementary file 1 [file ijms-21-07335-s001.zip › Sup_Figure 2_ijms.pdf]
